# A Novel Application of a Single-Entry Injection Technique for Non-surgical Penile Enlargement: A Case Report

**DOI:** 10.7759/cureus.83637

**Published:** 2025-05-07

**Authors:** Mohammed Chaudhry, Abd-Ur Raheem Chaudhry

**Affiliations:** 1 Aesthetics, Aiva Clinic, London, GBR

**Keywords:** dermal fillers, hyaluronic acid dermal fillers, non-surgical penis enlargement, penis enlargement, penis filler, penis fillers, penis filler techniques, penis rejuvenation, penis surgery, poly-l-lactic acid dermal fillers

## Abstract

Non-surgical penile enhancement using dermal fillers is a growing trend, yet traditional techniques often result in uneven volume distribution, poor integration, increased risk of filler migration and/or vascular complications. These limitations reduce aesthetic quality and patient satisfaction.

We introduce the Cylindrical Dartos-Buck Smooth (CDS) technique, a novel single-entry, cannula-based method designed for even filler distribution within the sub-Dartos/Buck’s fascial plane to achieve natural, uniform penile augmentation.

We present the case of a 29-year-old male who underwent the CDS technique with 15 mL of dermal filler via an 18-G blunt-tip cannula through a single mid-shaft entry point. Dermal filler was deposited in structured micro-droplets along the target fascial layer. At six-month follow-up, the patient demonstrated a 0.63-inch increase in girth, natural tactile feel, uniform volume distribution and no complications.

The patient and the injecting doctor completed a modified (adapted for penile enhancement) Global Aesthetic Improvement Scale (GAIS) to assess the results. The clinical GAIS score at six months was 2, and the patient GAIS score was 1, indicating an excellent clinical outcome and strong patient satisfaction.

The CDS technique provides enhanced control, superior contouring and improved safety compared to traditional penile filler approaches. Further studies are recommended to assess long-term outcomes and broader clinical utility.

## Introduction

Penile augmentation using dermal fillers has emerged as a promising non-surgical alternative to invasive procedures such as fat grafting, ligamentolysis and penile prosthesis implantation. Compared to traditional surgical techniques - many of which are associated with significant morbidity, high complication rates and variable long-term outcomes - filler-based enhancement offers a safer, more controlled approach with reduced downtime and improved patient tolerance [1,2].

The demand for minimally invasive penile enhancement has grown substantially in recent years, driven by improved filler technologies, advanced injection techniques and increasing social acceptability of male aesthetic procedures. Despite this rise in popularity, existing injection methods present notable limitations. Serial puncture needle injections are associated with uneven volumetric distribution, palpable nodularity and an increased risk of neurovascular trauma including ischaemic or occlusive complications [3,4].

Improper injection depth often results in the product being placed within the superficial subcutaneous tissue rather than the anatomically secure sub-Dartos/Buck’s fascial plane, leading to migration, irregular contouring and compromised structural stability [5]. 

Basic micro-cannula methods such as linear threading, fanning and cross-hatching often fail to provide consistent filler dispersion along the shaft, contributing to asymmetry, poor tactile integration and higher revision rates [6,7]. 

To overcome these challenges, we introduce the Cylindrical Dartos-Buck Smooth (CDS) technique, a novel single-entry single-plane filler injection method utilising a pulsed retrograde micro-droplet deposition strategy within the sub-Dartos/Buck’s fascial layer. This technique aims to deliver uniform volumetric expansion, enhanced filler stability and superior aesthetic outcomes with a reduced complication profile.

To the best of our knowledge, this case report details the first documented clinical application of the CDS technique and its early outcomes, positioning it as a potential new standard in non-surgical penile augmentation.

## Case presentation

A 28-year-old male in good general health presented for penile enhancement. Clinical assessment confirmed above-average penile dimensions in both flaccid (3.88 inches) and erect (5.3 inches) states, with no history of prior penile treatments. The patient had undergone neonatal circumcision and reported no functional concerns, seeking aesthetic improvement in girth and flaccid length.

Complete CDS technique

Aseptic Preparation

The procedural field was prepared under strict aseptic conditions, extending from the umbilicus to the knees to ensure comprehensive sterility. A dual antiseptic protocol was employed, incorporating surgical spirit and chlorhexidine gluconate solution, applied systematically to the mons pubis, inner and outer thighs, penile shaft and scrotum.

Anaesthesia

A dorsal penile nerve block was administered to provide complete regional anaesthesia. A total of 10 mL of 1% lidocaine was injected bilaterally at the 2 and 10 o’clock positions at the penile base, targeting the dorsal nerve and perineal branches. Aspiration preceded each injection to mitigate the risk of intravascular administration. Anaesthesia was confirmed via the absence of pain to light touch and needle prick.

Entry Point Access and Filler Injection Technique

An 18-G sharp needle was used to create a single pilot entry point at the mid-shaft. A total of 15 mL of dermal filler was administered. All injections were confined to the sub-Dartos/Buck’s plane, spanning from the penile base to approximately 3 mm distal to the circumcision scar.

Moulding

Following product deposition, the shaft was firmly massaged to facilitate even distribution. The patient was shown the post-procedure massage technique and instructed to continue daily for one week. The entry site was re-sterilised and sealed with a sterile low-profile dressing.

Dermal Filler Composition and Properties

The dermal filler chosen for this indication was aiva® Hybrid-Ultimate, a proprietary formulation developed by the authors specifically for penile augmentation. It consists of micronised poly-L-lactic acid (PLLA) microspheres suspended in a densely cross-linked, high-viscosity hyaluronic acid (HA) gel.

This dual-phase matrix delivers both immediate and long-term tissue enhancement. HA provides instant volume and shape, while PLLA induces gradual neocollagenesis, promoting firm, elastic collagen formation that mimics the natural biomechanics of penile tissue in both flaccid and erect states [8].

The microparticle dispersion is engineered to integrate uniformly within the tissue, reducing the risk of nodule formation or migration. The rheological profile of the HA matrix ensures ideal viscoelasticity for prolonged filler durability and tactile naturalness within the Dartos-Buck fascial interface [8].

Results

**Figure 1:**
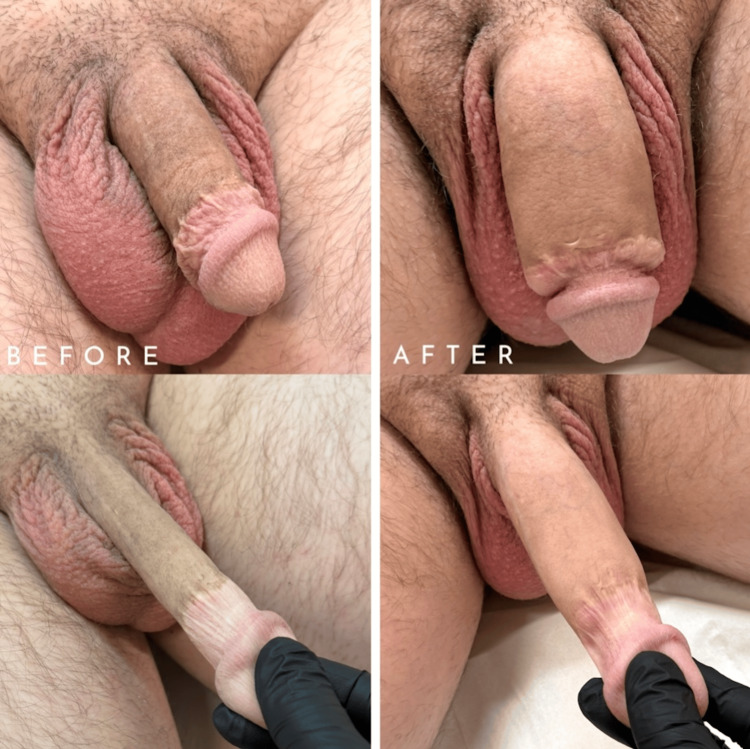
Side-by-side comparison before and six months post-treatment with 15 mL Hybrid-Ultimate filler

A 0.63-inch gain was achieved in flaccid girth (now 4.51 inches), which carried forward to erect girth gain (5.93 inches) (Figure 1). At six months post-treatment, the clinician assessed the patient as ‘Much Improved’ (GAIS score 2), noting substantial girth enhancement, symmetry and natural feel. The patient self-reported a GAIS score of 1 (Very Much Improved) with increased confidence and satisfaction (Table 1).

**Table 1:** Global Aesthetic Improvement Scale (GAIS) adapted for penile enhancement

Score	Description
1	VERY MUCH IMPROVED - Significant aesthetic and functional improvement; natural shape, symmetry and patient satisfaction
2	MUCH IMPROVED - Clear positive change; improved girth and appearance with minor asymmetry
3	IMPROVED - Noticeable improvement but with slight concerns (minor lumps, less symmetry)
4	NO CHANGE - Minimal or no visible difference
5	WORSE - Aesthetic or functional concerns post-treatment

## Discussion

We present the CDS technique as a superior alternative to conventional methods for non-surgical penile augmentation. This approach enhances both safety and efficacy, while addressing key limitations observed with traditional filler techniques [8]. 

**Figure 2:**
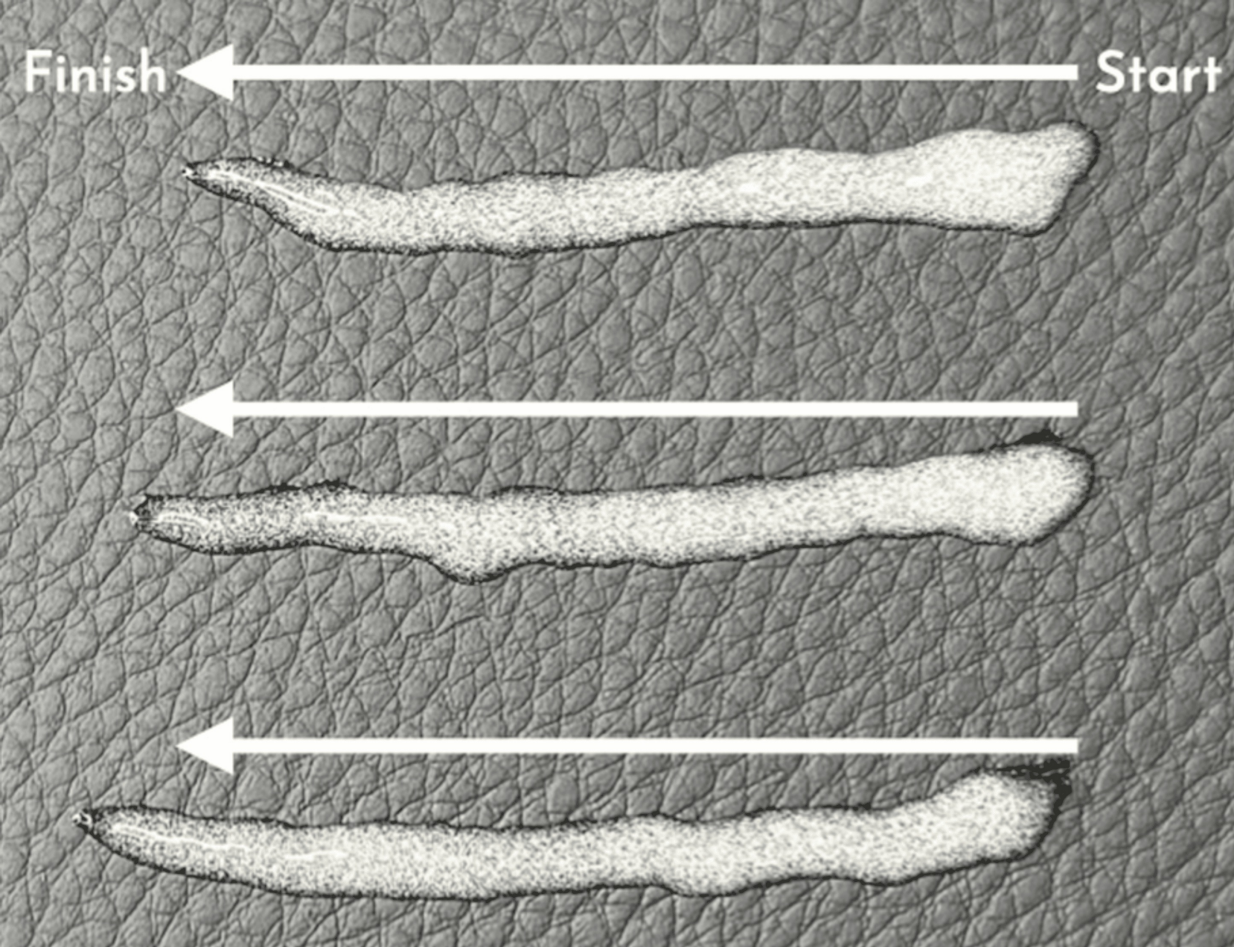
A visual representation of retrograde linear threads Image Credit: Mohammed Chaudhry and Abd-Ur Raheem Chaudhry

The widely used retrograde linear threading technique (Figure 2), although commonly practised, poses several challenges. By depositing filler in a continuous linear plane, this method leaves the material vulnerable to displacement under the dynamic contractile forces of penile tissue. As a result, patients frequently present with nodularity, uneven distribution and palpable irregularities - all of which compromise aesthetic harmony and functional outcomes [4,6,7]. 

**Figure 3:**
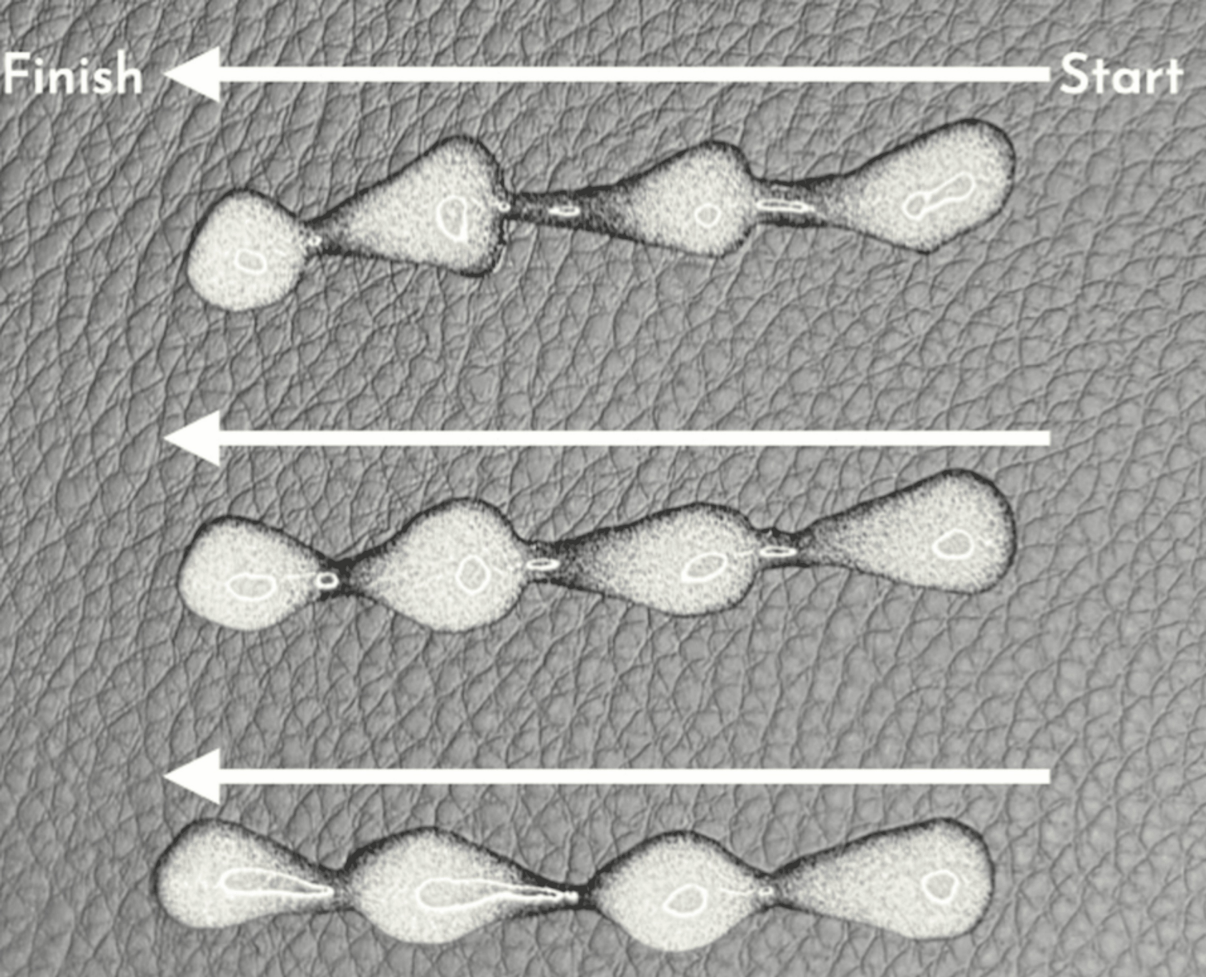
A visual representation demonstrating linear threads with pulsed microdroplet 'beads' of filler Image Credit: Mohammed Chaudhry and Abd-Ur Raheem Chaudhry

In contrast, our pulsed retrograde microdroplet bead technique significantly improves filler integration and stability (Figure 3) by placing larger microdroplet 'anchor points' intermittently along the retrograde path. The technique allows for three-dimensional dispersion while resisting migration. This results in smoother, more natural contours, enhanced volumetric symmetry and reduced risk of complications.

**Figure 4:**
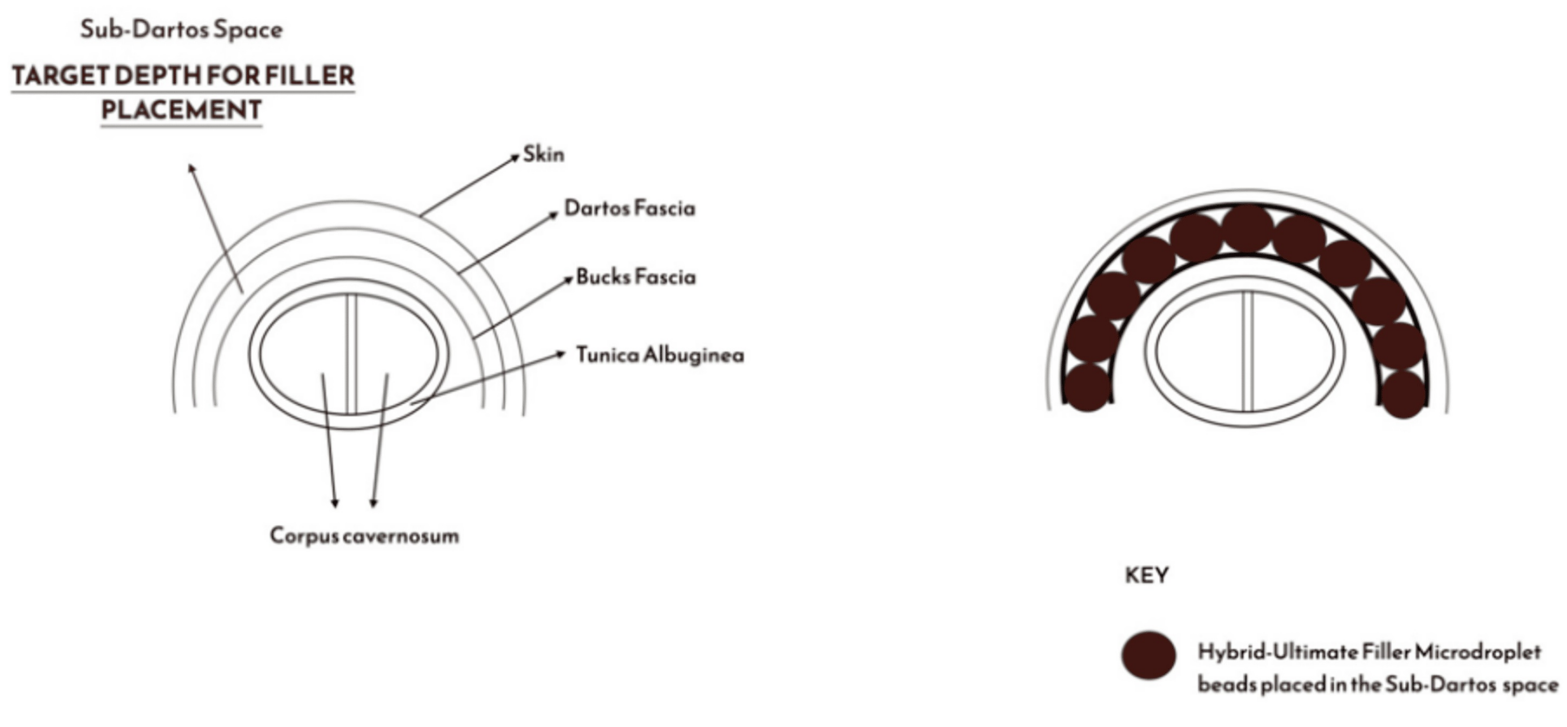
A cross-sectional anatomical schema illustrating the optimal filler plane for Hybrid-Ultimate penile enhancement The sub-Dartos space, between Dartos and Buck’s fascia, is identified as the safest, most effective layer for structural support and vascular preservation. Image Credit: Mohammed Chaudhry and Abd-Ur Raheem Chaudhry

Additionally, the introduction of a hybrid filler formulation, combining HA with PLLA, further advances the aesthetic and structural outcomes. HA contributes immediate volume and hydration, while PLLA acts as a collagen-stimulating scaffold, supporting the formation of dense penile collagen fibres during neocollagenesis in the sub-Dartos space (Figure 4).

This synergy not only improves long-term retention and tissue integration but also enhances shaft firmness and rigidity - key parameters for both flaccid and erect states [3-5]. Overall, the CDS technique, paired with a hybrid HA-PLLA formulation, represents a significant evolution in penile filler procedures. It offers a more predictable, stable and natural outcome, optimising both patient satisfaction and long-term structural integrity.

Limitations and considerations

Technical Expertise Required

The CDS technique necessitates a high level of anatomical understanding and advanced cannula handling skills. The sub-Dartos plane is a delicate and anatomically specific space that demands precise depth control to avoid superficial placement (which can lead to visible irregularities) or overly deep placement (risking proximity to neurovascular structures). As such, this technique should be reserved for experienced aesthetic practitioners with specific training in male genital anatomy and advanced filler techniques.

Individualised Approach Is Essential

Penile anatomy exhibits significant inter-individual variation in both shaft length, skin mobility and fascial thickness. A one-size-fits-all approach is therefore inappropriate. The volume of product used, filler concentration, depth of injection and entry point selection must be customised to each patient’s morphology and their specific aesthetic or functional goals. This consideration is critical for achieving optimal symmetry, natural proportions and consistent long-term outcomes.

Long-Term Data Still Required

While short-term outcomes with the CDS technique and HA-PLLA hybrid filler formulation appear promising in terms of patient satisfaction, aesthetic contour and tactile smoothness, comprehensive long-term data remain limited. Future studies should include larger patient cohorts with follow-up beyond 12-24 months to assess filler reabsorption timelines, collagen persistence and potential for delayed complications. In addition, histological evaluation of collagen density and tissue remodelling would offer valuable insight into the regenerative efficacy of this technique.

## Conclusions

The CDS technique represents a significant advancement in non-surgical penile augmentation. By combining a strategically layered microdroplet deposition method with a hybrid filler formulation of HA and PLLA, this approach offers enhanced filler integration, structural support and long-term aesthetic harmony. Compared to conventional linear threading methods, the CDS technique reduces the risk of migration, nodularity and irregular distribution while promoting natural tissue contour and firmness. The inclusion of PLLA introduces a regenerative element through collagen bio-stimulation, further improving both the durability and bio-mechanical quality of the results.

While initial outcomes are promising, further long-term data and multi-centre studies are encouraged to validate the reproducibility, longevity and safety profile of this novel approach. Nevertheless, the CDS technique, when performed by skilled practitioners, holds considerable promise as a next-generation standard in penile enhancement, merging functional precision with aesthetic finesse.
